# T Cell Repertoire Analysis as a Molecular Signature of the Spectrum of T-LGL Lymphoproliferative Disorders: Tracing the Literature

**DOI:** 10.3390/cimb47040264

**Published:** 2025-04-08

**Authors:** Evangelia Stalika, Ioannis Tsamesidis

**Affiliations:** Department of Prosthodontics, School of Dentistry, Faculty of Health Sciences, Aristotle University of Thessaloniki, GR-54124 Thessaloniki, Greece; johntsame@gmail.com

**Keywords:** T-LGL lymphoproliferative disorders, T cell repertoire analysis, hematological entities

## Abstract

CD3^+^ CD8^+^ CD57^+^ mono-, oligo-, and poly-clonal expansions, both idiopathic and clinically related diseases, including as autoimmunity, viral infections, post-transplant, and hematologic malignancies, can cause T large granular lymphocyte (T-LGL) lymphoproliferative disorders. It is yet unknown if this variability is a result of a dynamic process of cytotoxic T cell responses to exoantigens and autoantigens. The major aim of this review is to gather evidence from the literature in order to further highlight the possible pathogenetic mechanism that may underly the above clinical entities. Major research findings include the following: (i) pronounced skewing of the TRBV repertoire; (ii) existence of more than one immunodominant clonotype; (iii) persistent clonotypes in different timepoints albeit with fluctuating frequencies (clonal drift); and (iv) shared (‘public’) clonotypes between cases and the public databases, further suggesting a limited number of antigens implicated in pathogenesis of T-LGL cases. However, there is no clear distinction between polyclonal, oligoclonal, and monoclonal T-LGL lymphoproliferative conditions; rather, the progression from a polyclonal cytotoxic response to the emergence of T-LGL leukemia is slow. In the ontogeny and evolution of T-LGL leukemia, repertoire limits, public clonotypes, and clonal drift all clearly show selection by limited (perhaps shared) antigens.

## 1. Introduction

Molecular analysis of the T cell receptor repertoire in the study of hematological diseases with immune pathogenesis is very widespread, especially in the context of clonality detection. The concept of oligoclonality in the context of cellular immune responses is based on the presence of immunodominant T cell clones in the cellular subpopulations under analysis. Particularly for hematological research, molecular analysis of the T cell receptor repertoire and detection of the dominant clonotype can be applied as a diagnostic tool, offering new research perspectives in a number of hematological diseases [1,2].

Data from a series of studies belonging to the myeloid deficiency syndromes, such as T Cell large granular lymphocytic leukemia (T-LGLL) and myelodysplastic syndromes (MDS), suggest the presence of autoimmune processes in the context of the development of oligo/monoclonal cytotoxic T lymphocyte populations as the basis of myeloid failure. All relevant studies converge on the role of cytotoxic T lymphocytes in the context of a specific immune response. Additionally, it is worth noting that chronic neutropenia indicates a clinical landmark of T-LGL lymphoproliferative disorders, being present in the majority of T-LGLL cases [3]. Yet, the exact cause of T-LGL-related neutropenia remains unknown, and a number of processes, such as humoral abnormalities, bone marrow infiltration/substitution, and cell-mediated cytotoxicity, may contribute to its pathophysiology.

The present study aims to understand the pathogenesis of T-LGL lymphoproliferative disorders through a thorough study of the T cell receptor repertoire focusing on the literature data in different clinical entities. The advent of advanced next-generation sequencing technologies offers such depth of analysis of the T cell repertoire that it may lead to a revision of our notions of clonality and the composition of immune repertoires, overcoming the inherent weaknesses of Sanger sequencing.

## 2. Methods

Search methodology: A comprehensive literature search was conducted using the databases PubMed, Scopus, and Google Scholar. The literature review’s two main keywords were “T cell lymphocyte” and “haematology”. Then, the previously specified search parameters were limited to results that contained the term T-LGL lymphoproliferation. Additionally, the following pre-specified search terms were used to examine articles on T-LGL leukemia and myeloid failure syndrome, as well as the allogeneic hematopoietic cell transplantation: T cell lymphocyte AND hematology, T cell lymphoproliferation AND T-LGL leukemia, T cell lymphoproliferation AND myeloid failure syndrome, T cell lymphoproliferation AND allogeneic hematopoietic cell transplantation. The search was limited to the English language and the time of literature data collection and interpretation was from June to December 2024.

## 3. Results

### 3.1. Spectrum of T-LGL Status and Associations with Other Disorders

CD3^+^ CD8^+^ CD57^+^ T large granular lymphocyte lymphoproliferative disorders (T-LGL LPD) may be either idiopathic or develop in the context of viral infections, autoimmune disorders, after organ transplantation or allogeneic hematopoietic cell transplantation, as well as in patients after receiving anti-CD20 monoclonal antibody for the treatment of B lymphoma [4,5,6,7,8,9,10,11,12].

**Figure 1:**
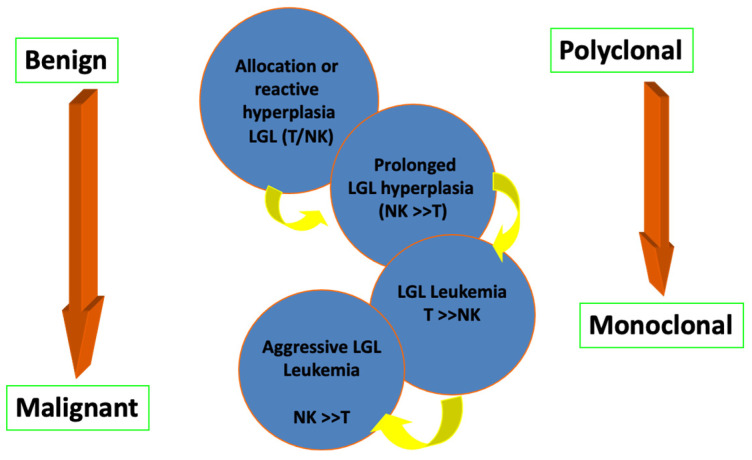
Spectrum of T-LGL disorders indicating the transition of Benign to Malignant entities and the progress from Polyclonal to monoclonal status (shown by arrows) (created by the authors, 2024).

They are divided into reactive and monoclonal, transient (<6 months) and chronic (>6 months) [13,14,15,16] (Figure 1). There is considerable overlap in the spectrum of clinical manifestations and laboratory findings between monoclonal (such as T-LGL leukemia) and reactive lymphoproliferative disorders from cytotoxic T lymphocytes which, as mentioned above, coexist with various clinical conditions.

Particularly, numerous causes, such as splenectomy, HIV infection, other viral infections, allogeneic stem cell transplantation, and solid organ transplantation, are linked to benign, reactive increases in LGLs [17,18]. Additionally, some older adults have persistent clonal expansions of CD8^+^ T-cell subsets, which do not progress to T-LGL leukemia [19]. Furthermore, T-LGL leukemia following allogeneic bone marrow (BM) transplantation has been documented. Though Epstein–Barr virus (EBV) infection was not detected in any of the cases, it was hypothesized that leukemia might have been caused by an antigenic reaction to cytomegalovirus infection or perhaps graft vs. host disease [20].

However, the implicated mechanisms that may underly LGL disorders remain unclear. Research evidence supports that malignant T-LGLs exhibit several characteristics of antigen-activated effector T cells, such as the presence of perforin, granzyme, and Fas ligand. Microarray technology has shown that in LGL leukemia, numerous genes related to cytotoxic functions are upregulated, such as granzymes, cathepsin, calpain, perforin, and caspase-8, displaying a specific pattern similar to that observed in activated cytotoxic T cells [21]. The specific antigen that stimulates these T cells remains unidentified, but serological evidence has shown that certain patients possess antibodies against proteins similar to those of human T lymphotropic virus I antigens, implying that a retroviral infection might contribute to the activation mechanism [22].

#### 3.1.1. Lymphoproliferative Cytotoxic T Lymphocytes and Hematological Diseases

A particular subtype of chronic cytotoxic T lymphocyte lymphoproliferative disorders is T large granular lymphocyte leukemia (T-LGL leukemia). LGL leukemia is distinguished into CD3^+^ LGL leukemia, in which the cells express and recombine T cell receptor genes, and CD3-(NK)-LGL leukemia, in which the neoplastic cells do not carry clonal rearrangement of T cell receptor genes but express the marker CD56.

T-LGL leukemia with the CD8^+^ CD4^−^ phenotype is due to the development of cytotoxic T lymphocytes that are subject to intense antigenic stimulation and are characterized by disturbances in the mechanism of apoptosis [23]. T-LGL leukemia occurs in a wide age range (from 4 to 88 years) with a median age of 55 years, affects both sexes equally and has a mild clinical course. The clinical picture includes splenomegaly, hepatomegaly, rarely skin infiltrates and lymphadenopathy. One third of patients have rheumatoid arthritis and some patients have arthritis, splenomegaly and neutropenia, thereby resembling the manifestations observed in patients with Felty’s syndrome (FS), which is a rare clinical condition of rheumatoid arthritis (RA) characterized by neutropenia and splenomegaly [24,25]. The disease usually has a chronic course and patients remain asymptomatic for more than 5 years with a median survival of 10 years.

The causes leading to the onset of T-LGL leukemia remain to be clarified. There is evidence that chronic persistent antigenic stimulation or a disorder in the mechanism of T-LGL cell apoptosis, e.g., due to a mutation in the *STAT3* gene, may lead to the disease [14,25,26,27,28,29,30,31]. In addition, it is hypothesized that a retroviral infection may play a role in the disease, which is supported by the finding of antibodies against HTLV (Human T cell Lymphotropic Virus) proteins [32,33]. Dysregulation of some intracellular signaling pathways, e.g., Fas/FasL, P13K and MAPK/ERK, is also associated with the resistance to apoptosis exhibited by leukemic LGL cells [34].

#### 3.1.2. T-LGL Lymphoproliferative Disorders and Myeloid Failure Syndromes: What Is the Exact Association?

T-LGL lymphoproliferative disorders overlap with other myeloid failure syndromes (myelodysplastic syndromes, aplastic anemia, paroxysmal nocturnal hemoglobinuria), in which the presence of autoimmune oligo/monoclonal cytotoxic T lymphocyte populations has also been reported. Cytopenias appear to be manifested due to destruction of hematopoietic stem cells by activated T lymphocytes through the secretion of lymphokines (e.g., IFNα, TNF and IL2) and/or increased destruction in the periphery, e.g., by an autoimmune mechanism [14,25,27,35,36,37].

##### Chronic Idiopathic Neutropenia (CIN)

Myeloid deficiency syndromes include chronic idiopathic neutropenia (CIN), which is an acquired granulomatous disorder characterized by prolonged neutropenia, mild and asymptomatic clinical course [38,39,40], and affects mainly middle-aged women with the HLA-DRB1*1302 type [41]. The hematopoietic failure in CIN appears to be mainly due to Fas-antigen-expression-induced apoptosis of CD34^+^/CD33^+^ hematopoietic progenitors and secondarily to increased concentrations of pro-inflammatory and pro-apoptotic cytokines of macrophage stromal origin in bone marrow. Increased numbers of stimulated T lymphocytes in the peripheral blood and bone marrow of patients are also reported as a major pathogenic mechanism, supporting a cause of pathogenesis of immune origin [42,43,44].

CIN shows significant clinical overlap with other disorders that result in neutropenia and are associated with chronic clonal expansion of CD8^+^ T lymphocytes, e.g., T-LGL leukemia and Felty syndrome (FS), as well as myeloid failure syndromes. However, while *STAT3* gene mutations are particularly common in T-LGL leukemia and Felty syndrome and less frequently reported in myeloid failure syndromes, they are particularly rare in CIN, thus suggesting a distinct pathogenetic mechanism [31,45,46].

Previous immunogenetic studies in CIN patient samples offered evidence of antigen selection in the modulation of the T lymphocyte repertoire. In particular, analyses based on the methodology of flow cytometry and classical subcloning and Sanger sequencing revealed the existence of clonal expansions of T cytotoxic lymphocytes in the peripheral blood and marrow of CIN patients, with distinct patterns of clonality among T lymphocyte subpopulations. Specifically, an oligoclonal/monoclonal pattern was observed in CD8^+^ cells, whereas the CD4^+^ cell subpopulation showed a polyclonal pattern [47]. In addition, strong selectivity of the T cell receptor β-chain gene repertoire was reported, suggesting selection from a limited range of antigenic elements [48]. However, due to the inherent limitations of low throughput (Sanger sequencing), it was not possible to draw definitive conclusions.

#### 3.1.3. T-LGL Lymphoproliferative Disorders and Allogeneic Hematopoietic Cell Transplantation

T-LGL neoplastic lymphoproliferative disorders must be differentiated from reactive conditions associated with hyperplasia, transient or permanent, of “normal” CD8^+^ cytotoxic T lymphocytes. These conditions usually coexist with autoimmune disorders and, potentially, may give rise to T-LGL leukemia. In this context, the development of cytotoxic T lymphocytes after allogeneic hematopoietic cell transplantation (allo-HCT), a key therapeutic option in hematological malignancies, marrow failure syndromes and inherited diseases, particularly of the immune system, is common. The occurrence of poly- or monoclonal T-LGL cell outgrowths in patients after allo-HCT can either be asymptomatic [49] or accompanied by cytopenias and autoimmune manifestations [4]. The strong association with viral infections in many of these cases implicates chronic antigenic stimulation in the pathogenesis of the entity. In this case, the overall immunosuppression of patients after transplantation seems to allow for the development of a T-LGL clone, which, normally, would be under the control of the immune system [4,5,50]. These types of reactive T-LGL cell deployments are usually polyclonal.

### 3.2. New Insights of the T-LGL Lymphoproliferative Disorders in the Next-Generation Sequencing (NGS) Era

In the past decade, next-generation sequencing (NGS) technologies have emerged, offering an unprecedented, detailed perspective on the T and B cell receptor immune repertoire. Research conducted across a range of inflammatory disorders, immunodeficiencies, infections, and cancers has revealed significant alterations in clonality, gene usage, and biophysical characteristics of the immune repertoire, yielding valuable insights into the roles of adaptive immune responses in different diseases [51].

Next-generation sequencing technology provides new evidence reshaping our previous insights regarding the role of T cell receptor in the pathogenesis of a spectrum of clinical entities. In order to obtain a more comprehensive view into the role of T cell receptor in the pathogenesis of T-LGL lymphoproliferative conditions, we summarize the key aspects of up-to-date studies in Table 1.

## 4. Discussion

Cytotoxic T lymphocyte lymphoproliferative disorders may be chronic or transient and occur in the context of heterogeneous clinical entities, e.g., coexisting with viral infections, autoimmune disorders or developing after organ or allogeneic hematopoietic cell transplantation [6,7,8,10,12,15,16,59,60,61,62]. Disorders in the mechanism of T lymphocyte apoptosis are believed to lead to the development of T lymphoproliferative diseases [34,63,64,65]; however, the ontogeny of these diseases has not been fully elucidated. It is believed that chronic persistent antigenic stimulation or some perturbation in the mechanism of T-cytotoxic lymphocyte apoptosis maintains the persistence of clonal outgrowths and eventual disease manifestation [28,66,67]. The nature of the antigens involved remains to be determined.

In the above context, the analysis of T cell receptor gene rearrangements in these entities may provide clues of the role of antigen in pathogenesis and possibly disease progression, assuming that intense antigenic stimulation initially leads to polyclonal hyperplasia and, under as yet unknown conditions (which could include genetic predisposition), may result in clonal disease, e.g., T-LGL leukemia [68].

In the last decade, the advent of high-throughput sequencing methodology and its application to T cell receptor repertoire studies in a wide range of physiological and pathological conditions has revolutionized immunogenetics [10,69,70,71,72,73,74,75,76]. A major challenge for the scientific community is the standardization of the experimental procedure and the development of bioinformatics approaches specifically designed for the analysis of immunogenetic data derived from high-throughput sequencing methods.

In the present study, we collected and presented literature data from various clinical entities associated with CD3^+^ CD8^+^ CD57^+^ large granular T lymphocyte lymphoproliferative disorders (T-LGL). It is well known that these lymphoproliferative disorders are manifested in a variety of heterogeneous pathological conditions [6,7,60,61,62]. It remains unclear whether this heterogeneity reflects a dynamic, multifactorial T cytotoxic lymphocyte response process against auto- and/or exogenous antigens. Previous data from Sanger sequencing studies indicated the possible existence of antigenic stimulation in the development of T-LGL lymphoproliferative disorders. However, the experimental shortcomings of small-scale sequencing did not allow for the drawing of firm conclusions [48,59,77,78,79,80,81].

Patients undergoing allogeneic hematopoietic cell transplantation and manifesting clonal T-LGL lymphocyte depletions may be either asymptomatic or have concomitant cytopenias and autoimmune manifestations [46]. T-LGL cell outgrowths in patients undergoing allogeneic hematopoietic cell transplantation may be monoclonal [82], but monoclonality does not indicate malignancy and is associated with an aggressive clinical course and adverse manifestations.

In summary, the findings from the analysis of T cell receptor genes in all the clinical ontotopes reported suggest persistent antigenic stimulation [28,59,78,83]. The latter hypothesis is supported by the selective utilization of TRBV-TRBD-TRBJ genes within the general immune response context, indicating selection from a limited range of antigens [28,84,85,86]. Finally, although cytopenias in the context of T-LGL lymphoproliferative conditions are multifactorial in etiology, the results of [25,87] suggest that TR specificity likely determines the spectrum of clinical manifestations with very specific recognition and destruction of specific hematopoietic lines [86]. Overall, the selectivity of the repertoire, the presence of public clonotypes, and the long-lasting persistence of depleted clonotypes strongly suggest selection by a limited number of antigens in the development and possibly progression of T-LGL leukemia, indicating that the boundaries between poly-, oligo- and monoclonality in T-LGL lymphoproliferative disorders are not clearly delineated and the transition from a polyclonal cytotoxic response to the development of T-LGL leukemia is a dynamic process of transition from a polyclonal hyperplasia to clonal disease.

This study of the broad disease spectrum of T-LGL lymphoproliferative disorders was extended to the immunogenetic analysis of cytotoxic populations in CIN, a usually mild and asymptomatic myeloid failure syndrome with selective neutropenia [39].

The presence of increased numbers of activated T lymphocytes in the blood and bone marrow of patients with CIN supports the hypothesis of the involvement of mechanisms of immune principle in the pathogenesis of the disease [36,44,47,48]. Previous findings from the molecular analysis of TRBV-TRBD-TRBJ T cell receptor rearrangements in CIN by classical subcloning and Sanger sequencing methods indicated strong skewing of the T cell receptor β-chain gene repertoire, indicating strong selection by a common antigen in disease development and, possibly, progression [48]. However, the limitations of small-scale immunogenetic analysis did not allow for the drawing of firm conclusions. However, literature evidence revealed the findings of strong TRBV gene selectivity, selectivity in the combination of specific TRBV/TRBJ genes in CDR3 sequence-specific lengths, oligoclonality [88] and “convergent recombination” (“convergent recombination”), [89,90,91] further supporting the hypothesis above. The latter observation is further supported the fact that specific T cell receptor amino acid sequences of particular antigenic specificity may play a critical role in the pathogenesis of CIN. These receptors could be synthesized and expressed by different cells, encoded by different nucleotide sequences and independently selected [90,92].

Additionally, literature data highlighted the existence of “public” (“common”) clonotypes among patients and/or the public base, which removes the logic of chance and strongly suggests selection by a common antigen in the natural course of the disease [5,77,93,94,95].

CIN shows a similar clinical picture to other entities associated with clonal expansion of cytotoxic T lymphocytes, e.g., myeloid failure syndromes and T-LGL leukemia, and the low frequency of *STAT3* gene mutations in CIN patients and the absence of specific immunogenic characteristics of mutants versus unmutated patients imply that CIN is a distinct entity from the other T/NK diseases, although the spectrum of their clinical manifestations shows overlap [31,46,96].

Considering all of the aforementioned data, we ultimately come to the conclusion that systemic and molecular disorders define the complex etiology of T-LGL leukemia, which is thought to involve neoplastic, viral, and/or autoimmune mechanisms. A chronic lymphoproliferative illness, LGL leukemia can appear with a variety of clinical manifestations [97]. LGL is currently incurable and very difficult to control. Immunosuppressive medications are the usual treatment; however, they have a significant recurrence rate. With encouraging outcomes, recent advancements in our understanding of the molecular processes have created opportunities to investigate innovative therapy modalities. It takes a coordinated effort using carefully planned clinical trials directed by a comprehensive understanding of the disease’s pathophysiology to address the pressing unmet need to enhance survival [98].

Moreover, recent research data further highlighted the major role that might be played by molecules, such as TNF-α and IFN-γ, and by overproduction in LGLs, which can activate the Fas-L pathway and increase the generation of reactive oxygen species (ROS), which can directly harm the BM microenvironment and exacerbate neutropenia [99]. In the context above, in order to obtain a more comprehensive view into the pathogenetic mechanism that may underly T-LGL lymphoproliferative conditions, additional functional studies could be established trying to correlate oxidative stress biomarkers and T-LGL hematopoietic disorders [100].

Ultimately, it is well known that the molecular mechanism of immunosenescence in tumors plays a multifaceted role. It involves (i) a number of factors (such as glucose competition and cAMP), (ii) the display of aberrant T cell phenotypes, such as the upregulation of CD57 and the downregulation of CD27 and CD28, which are closely linked to malignant tumors, and (iii) genetic and pharmacological intervention (such as interleukin-7 recombination and NAD^+^ activation). Emphasizing the traits of immunosenescence and how it affects cancerous tumors and immunotherapy may help us decide how to treat cancers in the future using senescence-focused approaches [101].

## 5. Conclusions

CD3^+^ CD8^+^ CD57^+^ T large granular lymphoproliferative disorders (T-LGL), transient or chronic, may develop in the context of a variety of heterogeneous pathological conditions (e.g., viral infections, autoimmune disorders, after organ transplantation, etc.).

Data from immunogenetic analysis of the T cell receptor β-chain by low-scale sequencing methods (Sanger sequencing) provided evidence for the involvement of persistent antigenic stimulation, which may be associated with the pathogenesis of these entities. However, the inherent weaknesses of the experimental approach above did not allow for the drawing of firm conclusions.

The development of new-generation sequencing methods (NGS—next-generation sequencing) in the last decade offers a more complete picture of the T cell receptor repertoire and more generally of the composition of immune responses involved in the pathogenesis and possibly in the progression of T chronic lymphoproliferative diseases, revealing: (i) repertoire selectivity, (ii) the presence of public clonotypes, and (iii) longitudinal persistence of deployed clonotypes. These findings strongly suggest selection by a limited number of antigens in clonal expansion. The nature of the involved antigens needs further investigation by functional studies.

## Figures and Tables

**Table 1:** Summary of the key aspects of the up-to-date studies regarding new insights of the T-LGL lymphoproliferative conditions provided by the NGS data.

Clinical Entity	Conclusions	Reference
Cutaneous T cell lymphomas (CTCLs)—Mycosis fungoides	1. An increased proportion of malignant T cell clones found in the skin is notably linked to reduced progression-free survival and overall survival rates in patients diagnosed with CTCL. 2. High-throughput DNA sequencing of the TCRβ gene revealed that a tumor clone frequency greater than 25% serves as a significant predictor of disease progression and unfavorable survival outcomes for MF patients with localized skin disease.	[52]
Systemic Lupus Erythematosus (SLE) and Rheumatoid Arthritis (RA)	1. Autoimmune diseases could lead to an increase in specific CDR3 amino acid sequences. 2. Significant changes in TRBV, TRBJ, IGHV and IGHJ genes. 3. Improve the understanding of TCR and BCR repertoires’ features and suggest an immune response to the common autoantigens in SLE or RA patients, which could assist in the development of targeted biotherapy and the diagnosis of SLE and RA.	[53]
Cord blood transplantation	Observation of clonal expansions in cord blood transplantation group by pinpointing antigen “specific” sequences in samples through VDJdb. The presence of clonally expanded sequences that are “specific” to HIV-1 in clinically confirmed HIV seronegative samples reinforces the notion of significant TCR cross-reactivity throughout the repertoire.	[54]
TCR repertoire in cancer	1. Evaluate immune diversity, assisting with early-stage cancer diagnosis, treatment selection, and prognosis prediction. 2. Dynamic TCR repertoire analysis may serve as a useful indicator of cancer development and guide immunotherapy. 3. In gastric cancer, low diversity of the TCR repertoire within the tumor-adjacent mucosal tissue is associated with a poor clinical prognosis in patients. 4. In lung cancer, a higher TCR repertoire diversity in tumor tissues is associated with worse cancer outcomes, while patients with a higher TCR diversity in peripheral blood show longer progression-free survival (PFS).	[55]
In B-cell lymphomas (BCL)/Diffuse Large B cell Lymphomas (DLBCL)	In BCL, a restricted TR repertoire is found to be associated with a poor outcome in DLBCL treated without immune-checkpoint inhibition and in high-grade B-cell lymphomas.	[56,57]
T-LGL lymphoproliferative disorders	1. The TRB gene repertoire in patients with T-LGL lymphoproliferations is highly dependent on context. 2. Different patterns of clonality across various disease scenarios. 3. Significant temporal clonal changes, suggesting that T-LGL lymphoproliferations could serve as an epiphenomenon in the presence of other cancers, potentially reacting to tumor antigens, for instance.	[58]
